# Induction of spontaneous human neocentromere formation and long-term maturation

**DOI:** 10.1083/jcb.202007210

**Published:** 2021-01-14

**Authors:** Marina Murillo-Pineda, Luis P. Valente, Marie Dumont, João F. Mata, Daniele Fachinetti, Lars E.T. Jansen

**Affiliations:** 1 Department of Biochemistry, University of Oxford, Oxford, UK; 2 Instituto Gulbenkian de Ciência, Oeiras, Portugal; 3 Institut Curie, Paris Sciences et Lettres, Research University, Centre National de la Recherche Scientifique, Unité Mixte de Recherche 144, Paris, France

## Abstract

Human centromeres form primarily on α-satellite DNA but sporadically arise de novo at naive ectopic loci, creating neocentromeres. Centromere inheritance is driven primarily by chromatin containing the histone H3 variant CENP-A. Here, we report a chromosome engineering system for neocentromere formation in human cells and characterize the first experimentally induced human neocentromere at a naive locus. The spontaneously formed neocentromere spans a gene-poor 100-kb domain enriched in histone H3 lysine 9 trimethylated (H3K9me3). Long-read sequencing revealed this neocentromere was formed by purely epigenetic means and assembly of a functional kinetochore correlated with CENP-A seeding, eviction of H3K9me3 and local accumulation of mitotic cohesin and RNA polymerase II. At formation, the young neocentromere showed markedly reduced chromosomal passenger complex (CPC) occupancy and poor sister chromatin cohesion. However, long-term tracking revealed increased CPC assembly and low-level transcription providing evidence for centromere maturation over time.

## Teaser

Murillo-Pineda et al. report a chromosome engineering system for human neocentromere formation and characterize the first experimentally generated human neocentromere. Neocentromere formation promotes local H3K9me3 eviction and cohesin and RNA polymerase II recruitment. Long-term culture results in gradual maturation of the inner centromere.

## Graphical Abstract

**Figure:**
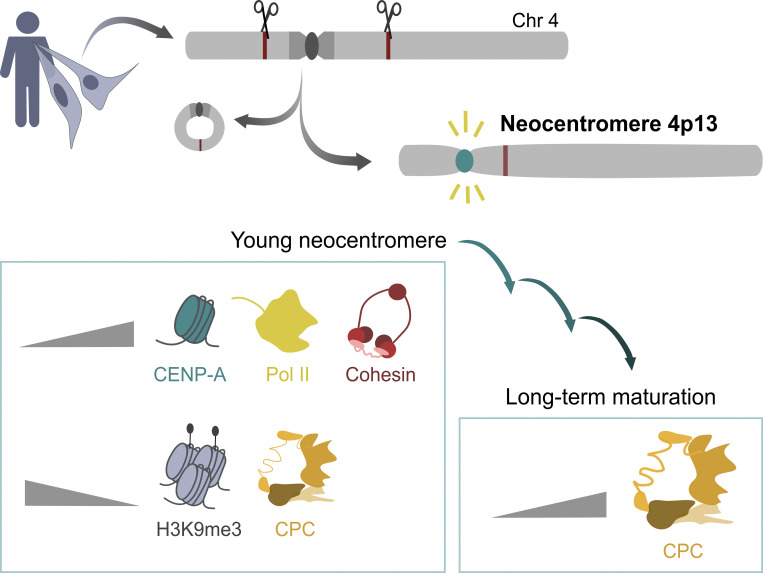

## Introduction

The centromere is the chromosomal locus defining the site for kinetochore assembly, which is essential for accurate chromosome segregation during cell division (Fukagawa and Earnshaw, 2014). In humans, centromeres are associated with α-satellite DNA, an array of head-to-tail repeats building up to more complex repeating patterns (Sullivan and Sullivan, 2020). A subset of these repeats (except on the Y chromosome) contains a motif for the binding of CENP-B, a centromeric protein that interacts with DNA in a sequence-specific manner (Earnshaw et al., 1987; Masumoto et al., 1989; Muro et al., 1992). However, despite contributing to centromere fidelity (Fachinetti et al., 2015; Hoffmann et al., 2016; Dumont et al., 2020; Hoffmann et al., 2020), centromeric DNA is not strictly required for centromere function, nor is it by itself sufficient to initiate centromere function (Earnshaw and Migeon, 1985). Instead, centromeres are primarily defined by specialized chromatin featuring the histone H3 variant CENP-A (Black and Cleveland, 2011). This epigenetic nature is exemplified in human neocentromeres (centromeres that vacated their canonical position at α-satellite DNA and repositioned to a naive locus not previously associated with centromere function; Voullaire et al., 1993; Tyler-Smith et al., 1999; Amor et al., 2004; Depinet et al., 1997). Approximately 100 neocentromeres have been isolated from human patients. Most are linked to genomic rearrangements leading to acentric DNA fragments (Marshall et al., 2008), indicating that centromere formation events are selected to stabilize the transmission of acentric DNA.

These naturally occurring phenomena have been the basis for several approaches designed to experimentally induce neocentromere formation by deletion of the endogenous centromere in different model organisms such as *Schizosaccharomyces pombe*, *Candida albicans*, *Cryptococcus deuterogattii*, or chicken cells (Ishii et al., 2008; Ketel et al., 2009; Shang et al., 2013; Schotanus and Heitman, 2020). These studies have identified features associated with centromere specification (reviewed in Murillo-Pineda and Jansen, 2020) but no universal commonalities. This may reflect species-specific differences in centromere organization and specification requirements. For example, neocentromere formation in fungi encounters compact genomes with few noncoding regions (Mohanta and Bae, 2015), and engineered chicken chromosomes contain specific smaller centromeres lacking repetitive DNA (Shang et al., 2010).

Here, we report a chromosome engineering system for neocentromere formation within the context of the complex human genome. We characterize the first spontaneously formed human neocentromere isolated in culture, advancing our current understanding of centromere formation, maturation, and stability.

## Results and discussion

### Isolation of an experimentally induced human neocentromere

**Figure 1.**
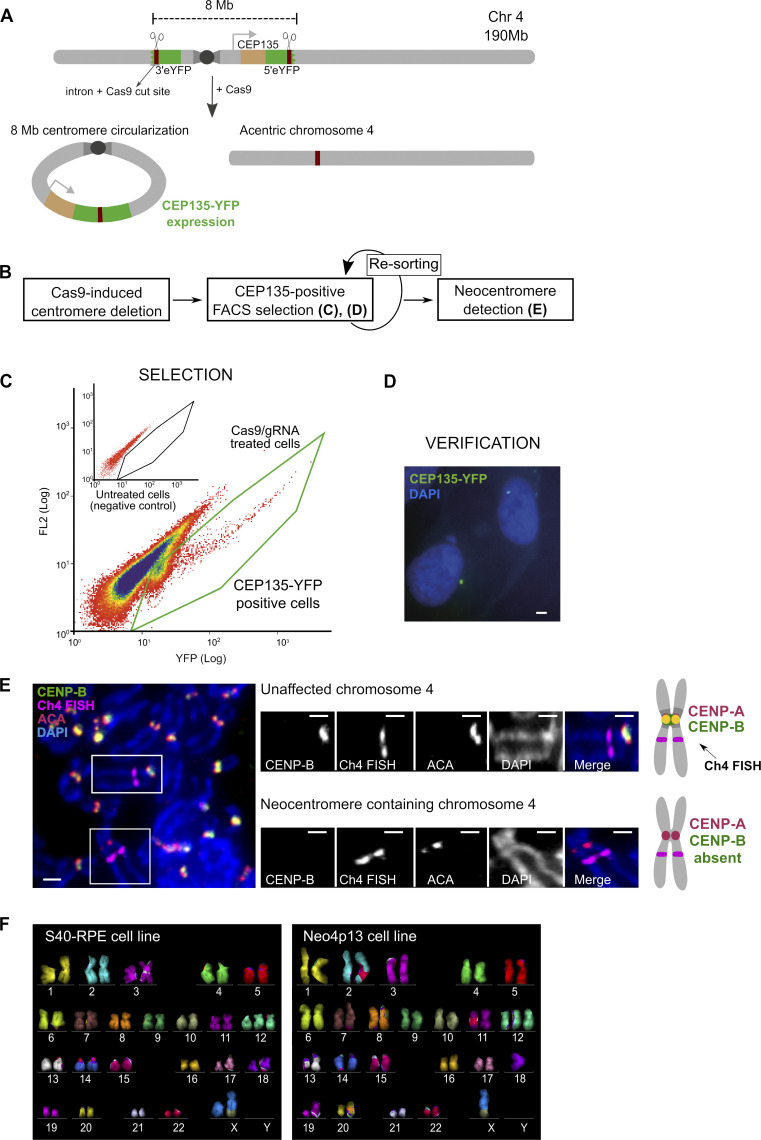
**Strategy for human centromere deletion and neocentromere isolation. (A)** A cassette carrying the 5′ portion of the eYFP was integrated on the 4q-arm in frame with CEP135 protein (light brown) and the 3′ portion on the 4p-arm, flanking the centromere. Cassettes carry a murine intron (dark brown) that acts as a gRNA target for Cas9. Upon Cas9 cleavage one possible outcome is the circularization of the centromere fragment (∼8 Mb) leading to reconstitution of spliced CEP135-eYFP. The acentric chromosome serves as template for neocentromere formation (see Fig. S1). **(B)** Experimental design for centromere deletion and neocentromere selection and detection. **(C)** Selection of CEP135-eYFP positive cells by flow cytometry (FL2, fluorescence channel 2 used for scatter and autofluorescence measurements). **(D)** Micrograph showing CEP135-eYFP foci indicative of centromere fragment circularization. Scale bar, 2 µm. **(E)** Micrograph of neocentromere detection by FISH-IF in mitotic spreads. The FISH probe identifies chromosome 4, ACA (CENP-A, CENP-B, and CENP-C) localizes to active centromeres, and CENP-B binds specifically to alphoid DNA, absent from neocentromeres. Insets display the two chromosome 4s from the image on the left. Scale bars, 2 µm. Diagram on the right represents the expected staining pattern to identify the neocentromere. **(F)** Karyotypes of the parental and neocentromere cell lines by mFISH. S40-RPE carries a preexisting translocation (X,10) and an extra copy of chromosome 12, present in all the clones analyzed. Chr4, chromosome 4.

**Figure S1.**
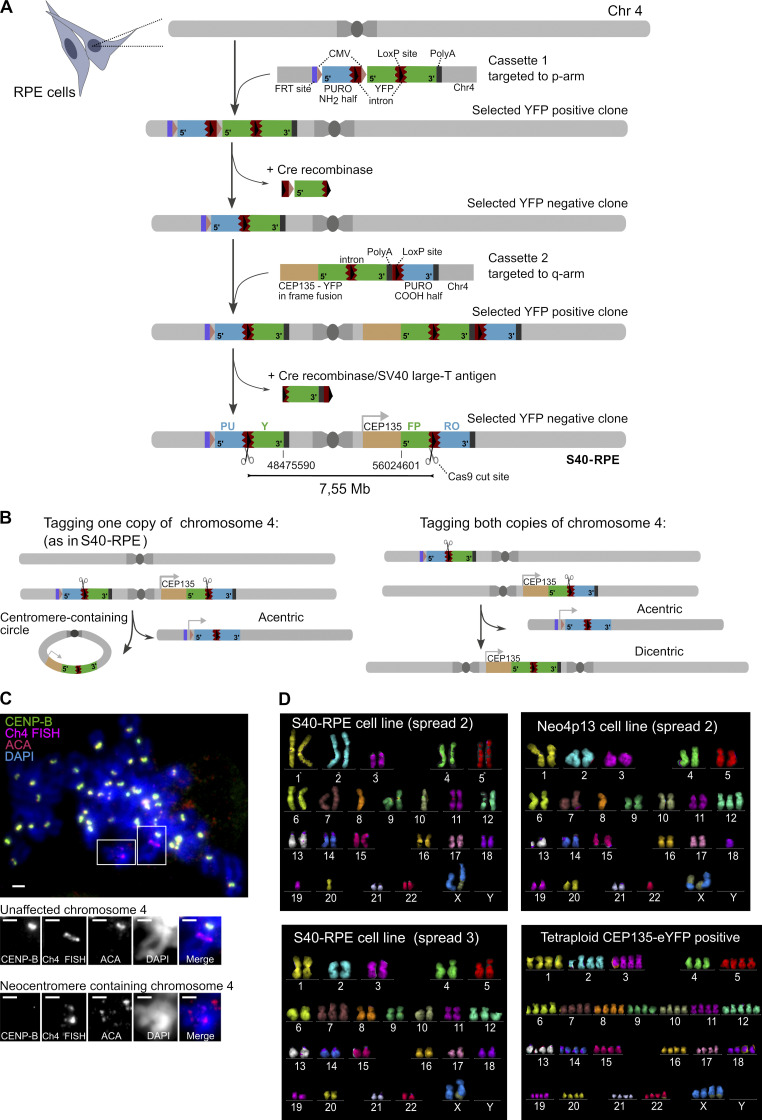
**Genomic architecture of strategy for centromere deletion and karyotype of neocentromere-containing cells and alternative survivors. (A)** Scheme of the sequential targeting steps and details of the targeting cassettes inserted into RPE cells flanking the centromere of chromosome 4. Constructs carry 5′ and 3′ fragments of the eYFP (green) split across the two genomic locations, separated by a mouse intronic sequence (brown) that is spliced efficiently (Lacy-Hulbert et al., 2001), and act as a target for gRNAs that will recruit Cas9 nuclease (scissors). Introns also carry a loxP site (black arrowhead). 5′ and 3′ fragments of a split Puromycin resistance gene (blue) are positioned downstream of the introns. Note that for the purposes of targeting the cassettes, a full-length eYFP gene was used to aid in fluorescence-based selection of targeted clones, followed by Cre-mediated removal of indicated gene fragments, ultimately resulting in the split eYFP arrangement flanking the centromere. Following Cas9 cut and repair, one possible outcome is the circularization of the centromere fragment (7.55-Mb region), leading to reconstitution of eYFP in frame with the centriole-associated protein CEP135. (Note that imperfect repair following the Cas9 cut is tolerated within the intronic region.) Conversely, the fusion of the acentric arms of the chromosome will lead to reconstitution of the Puromycin resistance gene driven from a cytomegalovirus (CMV) promoter (pink arrowhead) not used in this study. On the p arm, we also included an FRT (Flippase Recognition target) recombination site (purple), not used in this study. Indicated coordinates correspond to GRCh38 assembly. **(B)** Targeting of the p- and q-arm constructs from (A) result in two possible allele arrangements. Both possible scenarios (p- and q-targeted cassettes both inserted into the same chromosome 4 homologue [1] or in different ones [2]) lead to an acentric chromosome 4 formation after the Cas9 cut, serving as template for neocentromere formation. Based on mitotic spreads, multicolor karyotyping, and sequencing data, we deduced the S40-RPE parental cell line used in this study is of the scenario 1 type. **(C)** Early neocentromere detection by FISH-IF (performed as in Fig. 1 E) on the first CEP135-YFP–positive polyclonal population sorted 72 h after Cas9 electroporation and analyzed 3 d later. Scale bars, 2 µm. **(D)** Karyotype of indicated cell lines based on mFISH on different spreads, shortly after FACS-based isolation. Translocations and aneuploidies are detected in both the parental S40-RPE and Neo4p13 cell lines, possibly driven by SV40 expression. The tetraploid karyotype of a CEP135-eYFP–positive clone without an acquired neocentromere is also shown, indicative of single chromosome 4 loss. Chr, chromosome.

We developed a CRISPR-Cas–based approach in human retinal pigment epithelium (RPE) cells to select for spontaneous human neocentromere formation. We removed the endogenous centromere core and surrounding pericentromeric heterochromatin regions from one copy of chromosome 4, challenging cells with an acentric chromosome (Fig. 1 A). This large deletion avoids neocentromere formation at the endogenous locus due to residual CENP-A levels, as observed in chicken cells (Shang et al., 2013). Further, to aid in the detection of potential candidates for neocentromere formation, centromere deletion in our system results in the reconstitution of an enhanced yellow fluorescent protein (eYFP) gene in frame with the centriolar protein CEP135, allowing facile microscopy confirmation (Fig. 1, A and D; and Fig. S1 A). As Cas9 treatment results in the generation of double-strand breaks and consequent cell death, we express the oncogenic virus SV40 large T antigen in order to reduce the DNA damage response and maintain cell viability (parental cell line, S40-RPE).

We induced the Cas9 cut and sorted eYFP-positive cells after 72 h (0.01% to 0.15% of the population). Next, after a second round of sorting, we isolated single eYFP-expressing cells (Fig. 1, B–D), and mitotic spreads of cells with CEP135-eYFP signal were characterized by FISH and immunofluorescence (IF). We used a DNA probe to identify chromosome 4 and combined this with immunostaining for CENP-A and CENP-B to detect active centromeres and α-satellite DNA, respectively. We isolated a single clone with a putative neocentromere, detected as a locus positive for CENP-A/CENP-C (as marked by anti-centromere antibodies [ACAs]) but lacking CENP-B to indicate the absence of satellite DNA. This is, to our knowledge, the first experimentally induced human neocentromere on a naive chromosome, without pretargeting centromere components (Fig. 1 E). Neocentromere formation is relatively fast, detectable as early as we can isolate and analyze cells (∼6 d following Cas9 delivery; Fig. S1 C). Based on the chromosome shape in mitotic spreads and on PCR-Sanger sequencing, the two acentric fragments fused arm to arm near perfectly with a single-nucleotide deletion to create an otherwise-intact chromosome 4 (Fig. 1 E). We also confirmed the reconstitution of chromosome 4 and the maintenance of both copies without major structural rearrangements in the neocentromere-bearing cells by multicolor FISH (mFISH; Fig. 1 F). Note that in some cells, we observed aneuploidies of other chromosomes that appear unrelated to the neocentromere, as they were also present in S40-RPE parent cells (Fig. S1 D) and possibly relate to SV40 expression.

As our strategy allows the isolation of survivors after centromere deletion, we can uncover alternative survival paths. Indeed, we obtained four independent CEP135-eYFP–positive clones that lacked a neocentromere. We further karyotyped two of these by mFISH and uncovered that cells adapted by tetraploidization compensating for the loss of one chromosome 4 (Fig. S1 D). Therefore, unlike previously described for *S. pombe* (Ishii et al., 2008), chromosome fusion events involving the acentric chromosome 4 do not seem to be a major survival path in this case. Although calculating the neocentromere formation frequency is challenging, we can estimate an upper limit. We obtained a 0.05% median eYFP fluorescent population per experiment. Based on starting numbers, this represents ∼125,000 cells challenged with an acentric chromosome 4, giving rise to the neocentromere isolate. This indicates a frequency of 8 × 10^−6^, which is within the range observed for chicken cells (Shang et al., 2013).

### Reduced kinetochore size and strongly depleted inner centromere at nascent neocentromeres

**Figure 2.**
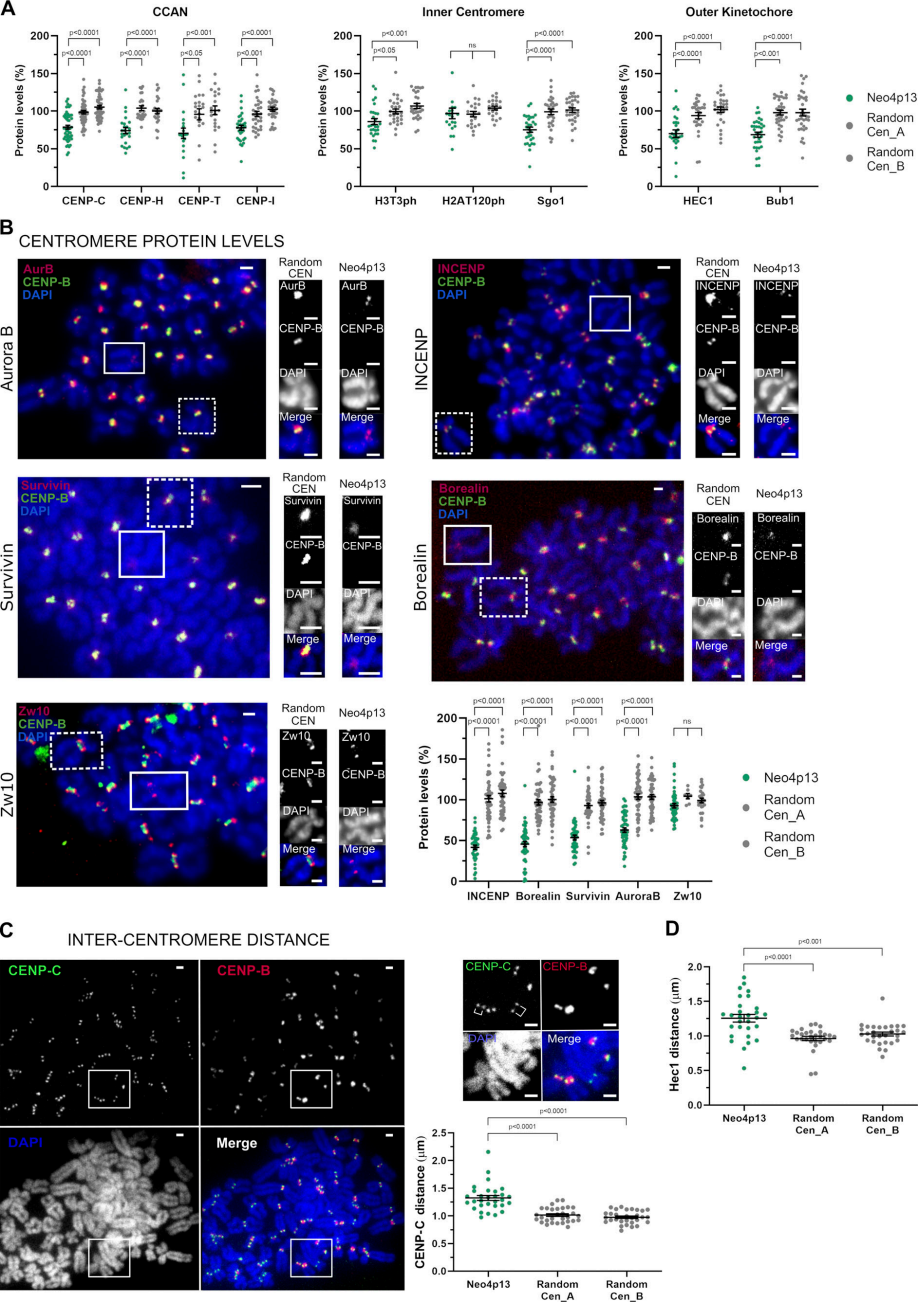
**Experimentally induced human neocentromere shows inner-centromere defects. (A)** Centromeric protein levels in the neocentromere (Neo4p13) compared with random endogenous centromeres. Quantification of mitotic spreads coimmunostained for indicated proteins and CENP-B. Mean and SEM of five (*n* = 50 spreads, for CENP-C) or three (*n* = 21–34 spreads, for all other proteins) independent experiments. P values based on one-way ANOVA with Tukey’s multiple comparison test. **(B)** CPC protein levels at Neo4p13 compared with random endogenous centromeres (Random CEN). Representative mitotic spreads coimmunostained for indicated CPC proteins and CENP-B. Zw10 outer kinetochore protein is included as a comparison to an unchanged reference protein. Insets show Neo4p13 and a random centromere equally scaled for visual comparison. Scale bars, 2 µm. Mean and SEM of five independent experiments (*n* = 45–52 spreads) analyzed as in A. **(C)** Intercentromere distance measured by coimmunostaining mitotic spreads for CENP-C and CENP-B. Quantification of distance (in micrometers) between the peak intensities of each CENP-C dot pair in one plane compared with equivalent pairs of random centromeres. Mean and SEM (*n* = 30 spreads) of three independent experiments. Scale bars, 2 µm. P values determined as in A. **(D)** Intercentromere distance based on Hec1 staining measured and analyzed as in C. Mean and SEM (*n* = 29 spreads) of three independent experiments.

Taking advantage of the lack of CENP-B staining as a marker for the neocentromere, we characterized neocentromere composition by performing immunostaining on mitotic spreads for different centromere and kinetochore components (Fig. 2, A and B). Unlike patient-derived neocentromeres that are isolated long after formation, our analysis focused on early passages following the isolation of the neocentromere. The majority of the constitutive centromere-associated network and outer kinetochore proteins are present at levels moderately, but significantly reduced relative to canonical centromeres (Fig. 2, A and B), as previously reported for CENP-A and CENP-C in a patient-derived neocentromere on chromosome 4 (Amor et al., 2004; Bodor et al., 2014; Fachinetti et al., 2015). In contrast, we found that levels of inner-centromeric chromosomal passenger complex (CPC) components are strongly reduced at the neocentromere (Fig. 2 B). Aurora B localization (although not levels) was shown to be affected in a patient-derived neocentromere (Bassett et al., 2010). Our finding that all CPC members are reduced suggest an overall impairment of CPC recruitment to neocentromeres. Interestingly, the two key chromatin marks involved in CPC recruitment, histone H3T3ph and H2AT120ph (Yamagishi et al., 2010; Wang et al., 2010; Kelly et al., 2010; Kawashima et al., 2007; Tsukahara et al., 2010; Kawashima et al., 2010), were close to or at normal levels at the neocentromere (Fig. 2 A). This suggests that another CPC recruitment factor is lacking or reduced at the neocentromere. Possibly, the CPC requires specific chromatin features, which may include DNA sequences features present within α-satellite DNA as recently suggested (Serena et al., 2020) or the structure of pericentric heterochromatin. In fact, HP1 is involved in CPC recruitment (Ruppert et al., 2018), and decreasing centromeric heterochromatin reduces CPC levels (Molina et al., 2016b). In addition, we detected a larger intercentromere distance when analyzing mitotic spreads stained with either a constitutive centromere-associated network or a kinetochore marker (Fig. 2, C and D), further indicating a defect in the inner-centromere structure. Combined, these findings suggest that CPC recruitment and possibly function depend on yet-undefined factors lacking at neocentromere sites, which may include HP1.

### The neocentromere spans a 100-kb CENP-A domain in a gene-poor region

**Figure 3.**
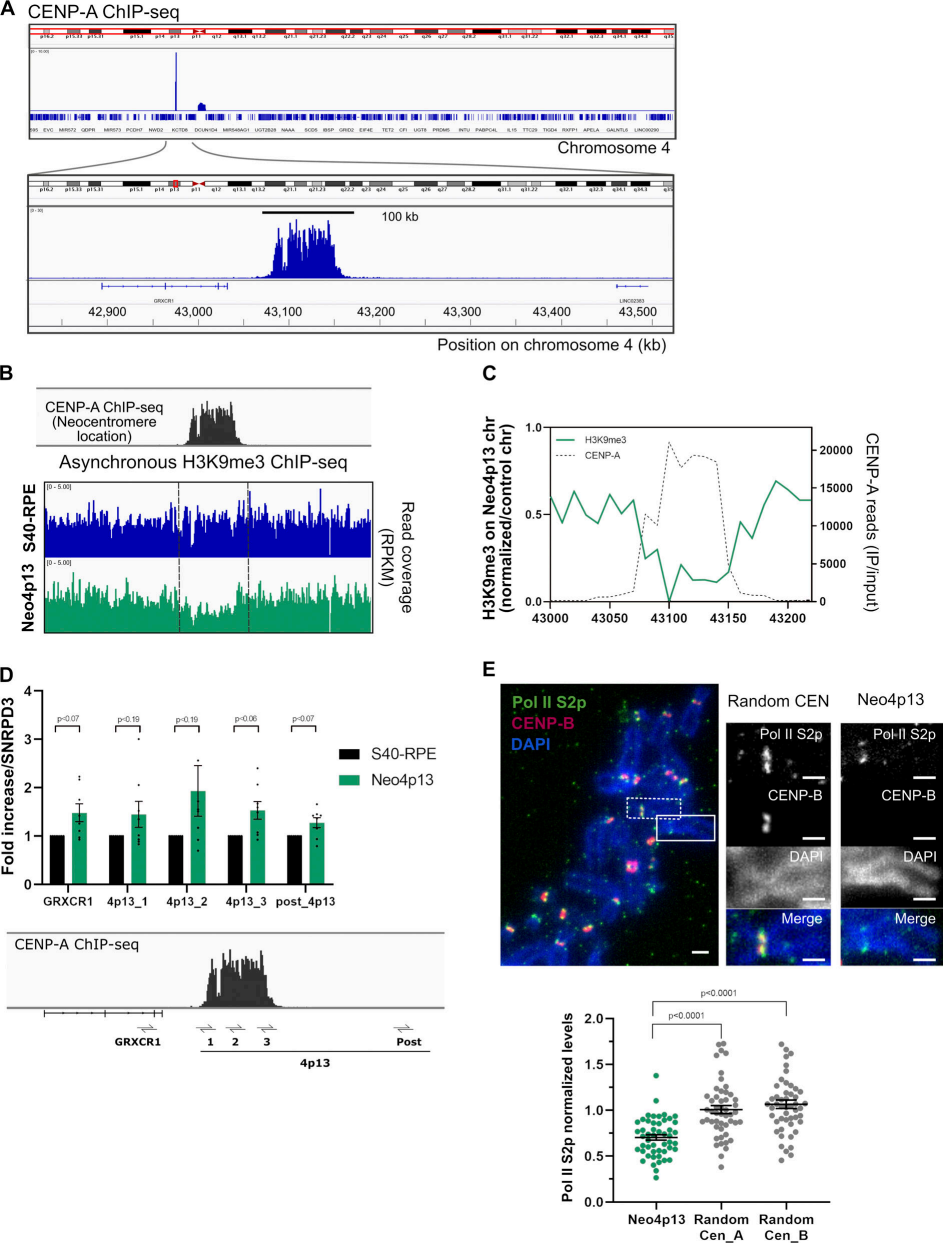
**Neocentromere spans 100 kb in a novel heterochromatic gene-poor region and recruits RNA Pol II upon formation. (A)** CENP-A occupancy along chromosome 4 analyzed by ChIP-seq. Below, blowup of neocentromere region spanning around 100 kb at the novel 4p13 location. **(B)** Genomic snapshot of quantitative ChIP-seq reads (RPKM, reads per kilobase per million) for H3K9me3 (asynchronous cells) plotted across the 4p13 location, before (S40-RPE) and after neocentromere formation (Neo4p13). **(C)** Estimated allele specific coverage of H3K9me3 on the 4p13 region (marked by CENP-A ChIP-seq, gray line) on the neocentromere-containing chromosome in Neo4p13 cells. Calculation is based on the assumption that the read coverage from the nonaffected chromosome 4 in Neo4p13 cells is equivalent to average coverage in S40-RPE cells (see methods). **(D)** Quantitative RT-PCR analysis of the 4p13 CENP-A domain and adjacent regions before (S40-RPE) and after neocentromere formation (Neo4p13). Primer locations are indicated below the CENP-A ChIP profile. Mean fold changes and SEM of eight independent experiments are plotted relative to S40-RPE and normalized against the SNRPD3 reference gene (Eisenberg and Levanon, 2013). Adjusted P values from a multiple *t* test analysis are shown. **(E)** Pol II S2p levels in Neo4p13 compared with random endogenous centromeres. Quantification of mitotic spreads as in Fig. 2 A. Scale bars, 2 µm. Mean and SEM of five (*n* = 50 spreads) independent experiments. P values based on one-way ANOVA with Tukey’s multiple comparison test.

Next, we mapped the precise genomic location of the nascent human neocentromere by native CENP-A chromatin immunoprecipitation and sequencing (ChIP-seq). We detected a single defined peak on chromosome 4 at position 4p13 (henceforth named Neo4p13), 6 Mb distal to the deleted centromere, in a region not previously described for any of the patient-derived neocentromeres (Marshall et al., 2008; Fig. 3 A). Neo4p13 spans a CENP-A domain of ∼100 kb, which is considerably smaller compared with canonical centromeres (Dumont et al., 2020) but within the range of patient-derived human neocentromeres (80–300 kb; Hasson et al., 2013; Alonso et al., 2010) or species containing native nonrepetitive centromeres such as horses (Nergadze et al., 2018; Purgato et al., 2015). The reduced size may explain the lower accumulation of most centromeric proteins tested compared with canonical centromeres (Fig. 2, A and B). Nevertheless, as canonical centromeres span larger domains, protein density might still be comparable.

### Neocentromere formation is driven by epigenetic mechanisms

**Figure S2.**
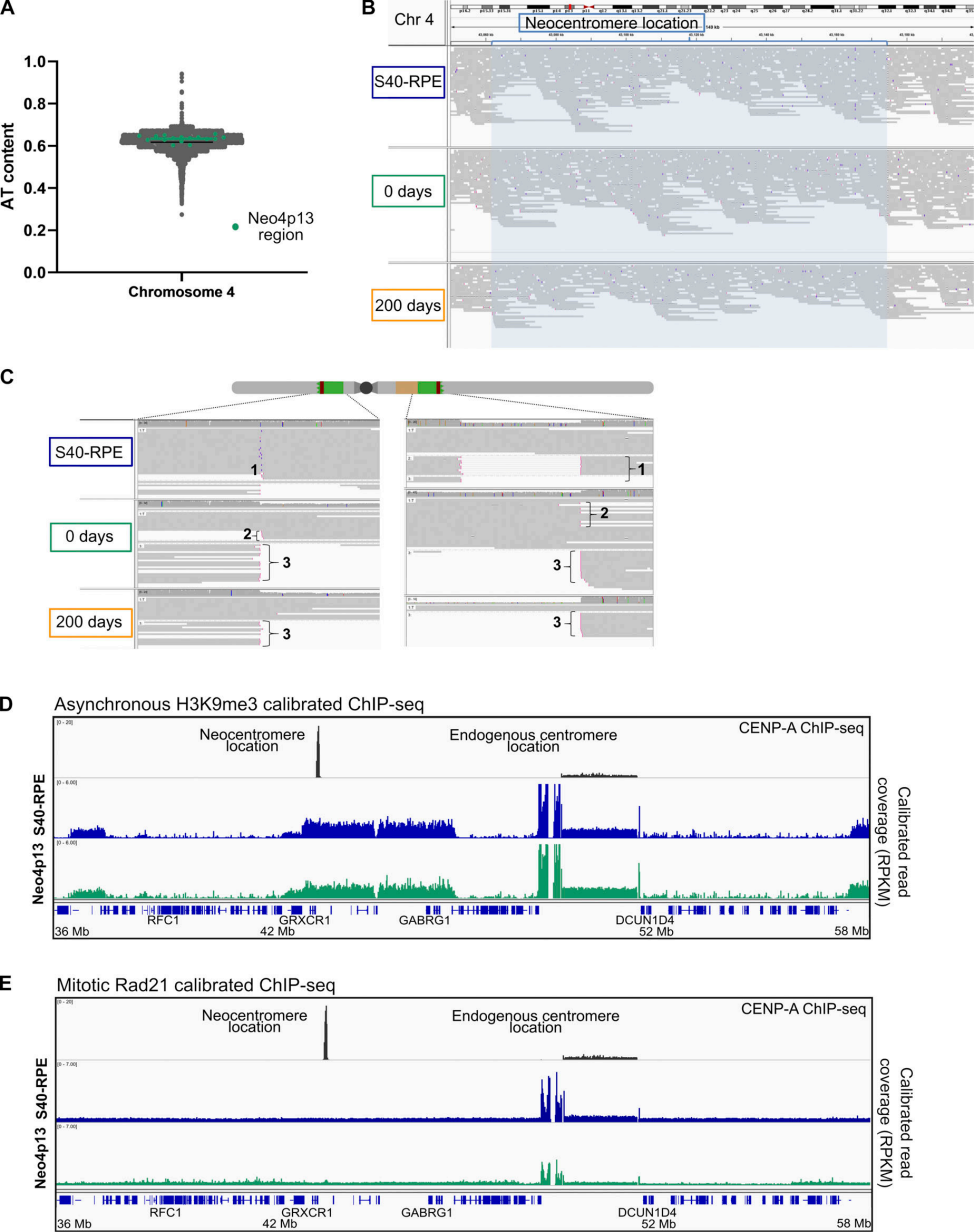
**Neocentromere formation is driven by epigenetic mechanisms. (A)** AT content along chromosome 4 (Chr 4)in 10-kb windows using isochore function from EMBOSS 5.0.0. Mean value for chromosome 4 (61.85%, black line) and overlay of the AT content at the neocentromere region (63.4%, green line). **(B)** Long-reads (12 kb average) from nanopore sequencing (PromethION) mapped against the hs37d5 human reference genome resulting in 93 Gb, 99 Gb, and 59 Gb of mapped data for the S40-RPE, Neo4p13 0 d and 200 d of culture, respectively. Neocentromere location snapshot is shown (blue shade) with purple bars indicating insertions larger than 10 bp. **(C)** Long-read coverage identified cassette integration sites in the control cell line (1). Pink ends indicate clipped reads, where at least 100 bp at the end of the read is either unmapped or maps to another location in the genome. We also detected reads supporting the centromere deletion in Neo4p13 at both time points (3) and reads from the excised circular centromeric DNA region (containing eYFP sequence) only at time 0 (2). **(D and E)** Genomic snapshot of calibrated ChIP-seq reads (RPKM, reads per kilobase per million) from H3K9me3 from asynchronous cells (D) and Rad21 from mitotically enriched cells (E), covering 22 Mb of chromosome 4, including the neocentromere location and the endogenous centromere region, before (S40-RPE) and after neocentromere formation (Neo4p13).

It has been proposed that neocentromeres form preferentially on AT-rich regions (Marshall et al., 2008). The 4p13 locus has an AT content slightly higher than the overall chromosome, although this is by no means unique and thus unlikely to explain centromere formation (Fig. S2 A). We further analyzed the locus for the presence of LINE elements, previously connected with a patient-derived neocentromere (Chueh et al., 2009), as well as for other DNA features. In general, we concluded that the neocentromere locus is not enriched in any particular DNA sequence or structure.

In addition, neocentromere formation could have been accompanied, or driven by, acquisition of novel DNA sequences. Indeed, canonical human centromeres are associated with repetitive DNA, and there is emerging evidence for an important contributing role for α-satellite DNA in centromere function (reviewed in Murillo-Pineda and Jansen, 2020). Moreover, work on de novo centromere formation using ectopic human artificial centromeres showed that neocentromere seeding can occur on rearranged DNA that acquired fragments of centromeric DNA, suggesting satellite DNA to be a driving force for centromere formation (Logsdon et al., 2019).

Unlike with patient-derived neocentromeres, we have access to the ancestral genomic state before neocentromere formation. Therefore, we sequenced the Neo4p13 line along with its parent to detect any DNA sequence acquisition that may have occurred. To overcome mapping difficulties associated to repetitive DNA, we performed long-read nanopore sequencing (PromethION) with average read lengths of 12 kb (Fig. S2 B and Materials and methods). Analysis of structural variants within the Neo4p13 region revealed no evidence of translocations or insertion of α-satellite sequences or any other change in DNA composition compared with S40-RPE with a 10-bp detection cutoff. Nanopore sequencing also confirmed the junction between the two arms of chromosome 4 as well as circularization of the excised centromere fragment (Fig. S2 C). In sum, detailed DNA sequence analysis indicates that the 4p13 neocentromere is a true epigenetic event.

### Neocentromere formation promotes H3K9me3 eviction and cohesin and RNA polymerase II (Pol II) accumulation

Neo4p13 originated in a gene-poor region with the nearest annotated gene GRXCR1, ∼45 kb from the CENP-A domain, which is not expressed in RPE cells (He et al., 2018). The apparent lack of transcription prompted us to determine the heterochromatin status in this region by analyzing the levels of H3K9me3. Heterochromatin has been shown to contribute to neocentromere specification in yeast and *Drosophila melanogaster* tissue culture cells (Ishii et al., 2008; Olszak et al., 2011). However, the significance of heterochromatin is unclear, as in *C. albicans*, chicken cells, human patients, or an in vivo *Drosophila* system, no such requirement is reported (Ketel et al., 2009; Shang et al., 2013; Alonso et al., 2010; Palladino et al., 2020). We found a broad domain of H3K9me3 enriched at the 4p13 locus before neocentromere formation, suggesting that in this case, heterochromatin has been permissive for centromere seeding (Fig. S2 D). Next, we characterized the heterochromatin status following neocentromere establishment and detected an inverse correlation for H3K9me3 levels within the CENP-A domain (Fig. 3 B). This suggest that CENP-A seeding competes with heterochromatin or that H3K9me3 is otherwise incompatible with CENP-A chromatin. ChIP analysis results in an aggregate signal from both chromosome 4 homologues. However, as only one carries the neocentromere, and assuming that the other homologue will remain unaltered, we can estimate H3K9me3 levels to be reduced by 78% at the Neo4p13 CENP-A site (Fig. 3 C). It has been shown that heterochromatin seeding can inactivate human centromeres (Nakano et al., 2008; Cardinale et al., 2009; Ohzeki et al., 2012). Possibly, H3K9me3 reduction may reflect transcriptional changes due to neocentromere formation, as centromeric transcripts and transcription have been shown to play a role in centromeric chromatin assembly and maintenance (reviewed in Perea-Resa and Blower, 2018). We assessed transcription by qRT-PCR with probes within and flanking the CENP-A domain. We detected up to a twofold increase in transcription at the 4p13 region, within and proximal to the CENP-A peak, in Neo4p13 (Fig. 3 D). This may indicate a low increase of transcription within the overall region. To explore this further, we determined whether actively transcribing RNA Pol II accumulates specifically in mitosis, as has been shown for canonical centromeres (Chan et al., 2012; Rošić et al., 2014; Molina et al., 2016b). Immunostaining of mitotic spreads revealed the presence of serine 2 phosphorylated polymerase II (Pol II S2p) at the Neo4p13 even at early passages following its isolation (Fig. 3 E). The levels of Pol II S2p are significantly lower relative to canonical centromeres, although the level scales with the overall smaller neocentromere size.

**Figure 4.**
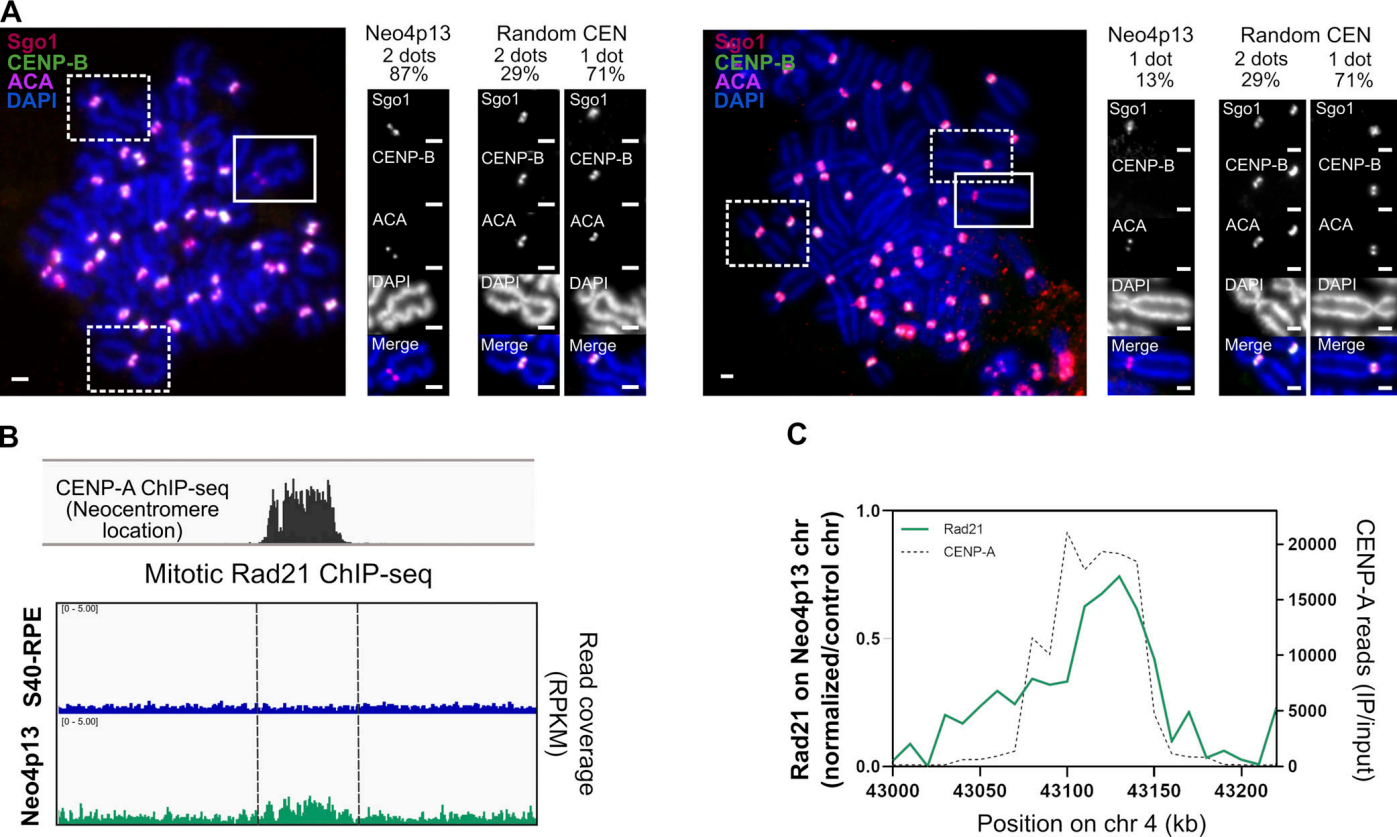
**Sgo1 localization at the neocentromere is kinetochore biased. (A)** Sgo1 localization in Neo4p13 compared with random endogenous centromeres. Representative mitotic spreads coimmunostained for Sgo1, ACA, and CENP-B. Insets show Neo4p13 and random centromeres with Sgo1 localization toward the kinetochores (two dots) or the inner centromere (one dot) and the quantification of each localization pattern (%) from three independent experiments (*n* = 50 spreads). P < 0.0001 (*t* test). Scale bars, 2 µm. **(B)** Genomic snapshot of quantitative ChIP-seq reads (RPKM, reads per kilobase per million) for Rad21 (mitotic cells) plotted as in Fig. 3 B. **(C)** Estimated allele specific coverage of Rad21 on the 4p13 region on the neocentromere-containing chromosome in Neo4p13 cells inferred as in Fig. 3 C. chr, chromosome; IP, immunoprecipitation.

Pol II transcription in mitosis at centromeres has also been shown to drive Shugoshin (Sgo1) from a kinetochore to inner-centromere localization (Liu et al., 2015). Nonetheless, despite the presence of Pol II at the neocentromere, we observe that Sgo1 localization is biased toward kinetochores (as revealed by two resolvable foci; Fig. 4 A). This could be a consequence of much lower transcription at the neocentromere compared with canonical centromeres. However, Sgo1 recruitment to the inner centromere also depends on the accumulation of cohesin, the large SMC protein–containing complex involved in sister-chromatid cohesion (Liu et al., 2013). To assess neocentromere cohesin levels, we performed Scc1 (Rad21) ChIP-seq in mitotically arrested Neo4p13 cells as centromere enrichment of cohesin is restricted to mitosis. Indeed, we were able to detect enrichment of cohesin specifically at the 4p13 site in a neocentromere-specific manner (Fig. 4, B and C), indicating that the nascent centromere has gained the ability to recruit or stabilize cohesin complexes. Nevertheless, cohesin levels appear to be low compared with endogenous centromeres (Fig. S2 E), possibly due to the lower levels of CPC proteins (Hengeveld et al., 2017). Moreover, this lower level of cohesin may affect the inner-centromere localization of Sgo1 at Neo4p13 and may explain the larger intercentromere distance (Fig. 2, C and D). In addition, condensin also contributes to inner-centromere structure and maintaining intercentromere distance (Hudson et al., 2003; Ribeiro et al., 2009; Samoshkin et al., 2009), although this was not directly assessed in this study. Combined, these findings suggest that acquisition of a proper inner-centromere structure is deficient following neocentromere formation.

### Stable propagation and chromatin maturation of experimentally induced neocentromeres

**Figure 5.**
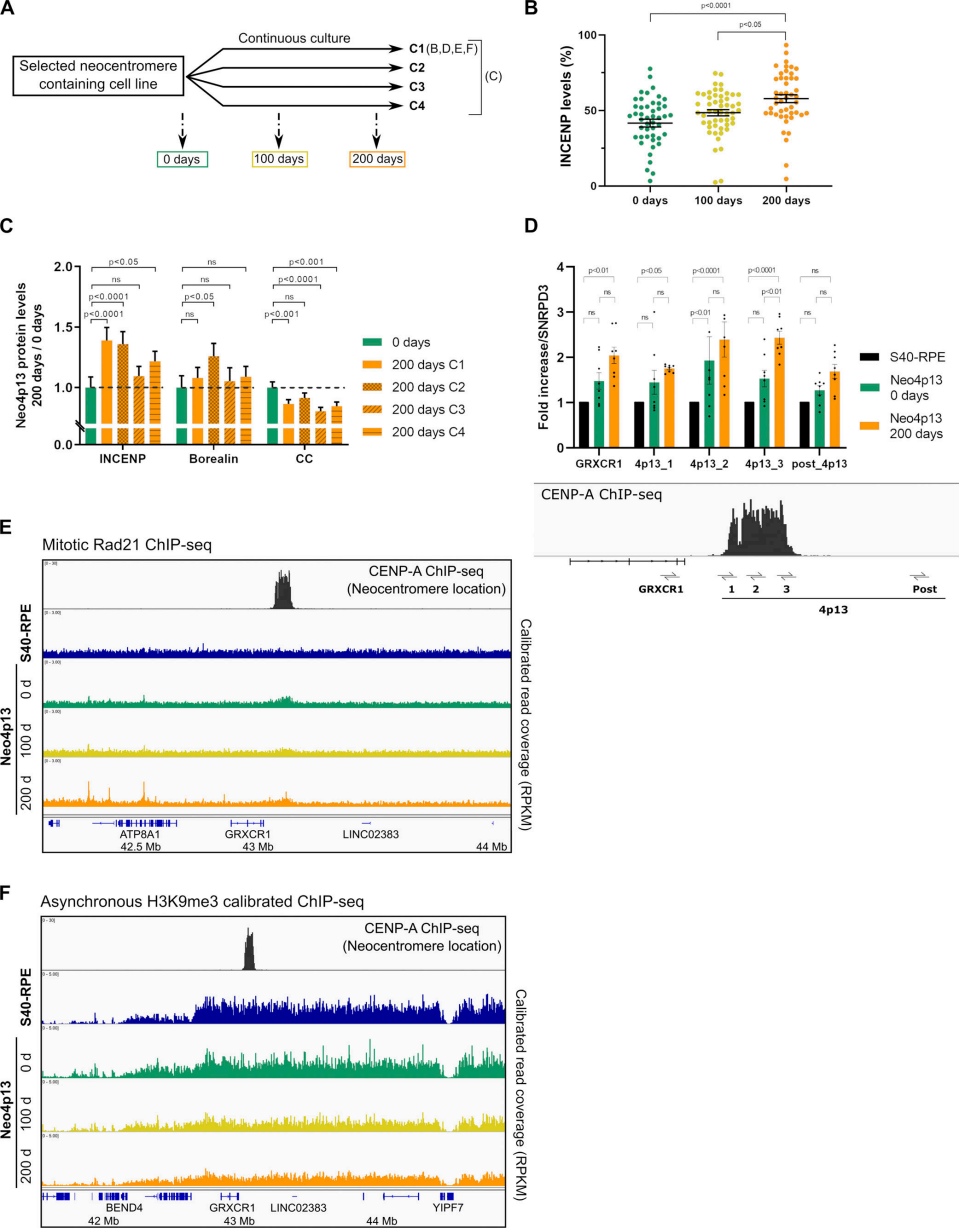
**Neocentromere formation and maturation promote changes in transcription, chromatin, and inner-centromere protein recruitment. (A)** Experimental design to propagate independent clonal populations (C, independent clones) and compare neocentromere status early after isolation and after adaptation through successive generations. **(B)** INCENP levels at Neo4p13 at different times points (0, 100, and 200 ds of continuous culture) determined as in Fig. 2 A. Mean and SEM of five independent experiments (*n* = 47–52 spreads). P values based on one-way ANOVA with Tukey’s multiple comparison test. **(C)** INCENP, Borealin, and CENP-C (CC) levels in different clones of Neo4p13 (C1–C4) after 200 d of continuous culture measured as in Fig. 2 A, normalized to protein levels at day 0. Mean and SEM of five independent experiments (*n* = 44–50 spreads). P values determined as in B. **(D)** Quantitative RT-PCR analysis of 4p13 CENP-A domain and adjacent regions before (S40-RPE) and at 0 and 200 d of continuous culture following neocentromere formation (Neo4p13) as in Fig. 3 D. Mean fold changes and SEM of eight independent experiments. P values from two-way ANOVA analysis are shown. **(E and F)** Neocentromere chromatin status at 0, 100, and 200 d of continuous culture measured by quantitative ChIP-seq. Genomic snapshot of 4p13 location showing Rad21 (mitotic cells; E) and H3K9me3 (asynchronous cells; F) normalized coverage.

**Figure S3.**
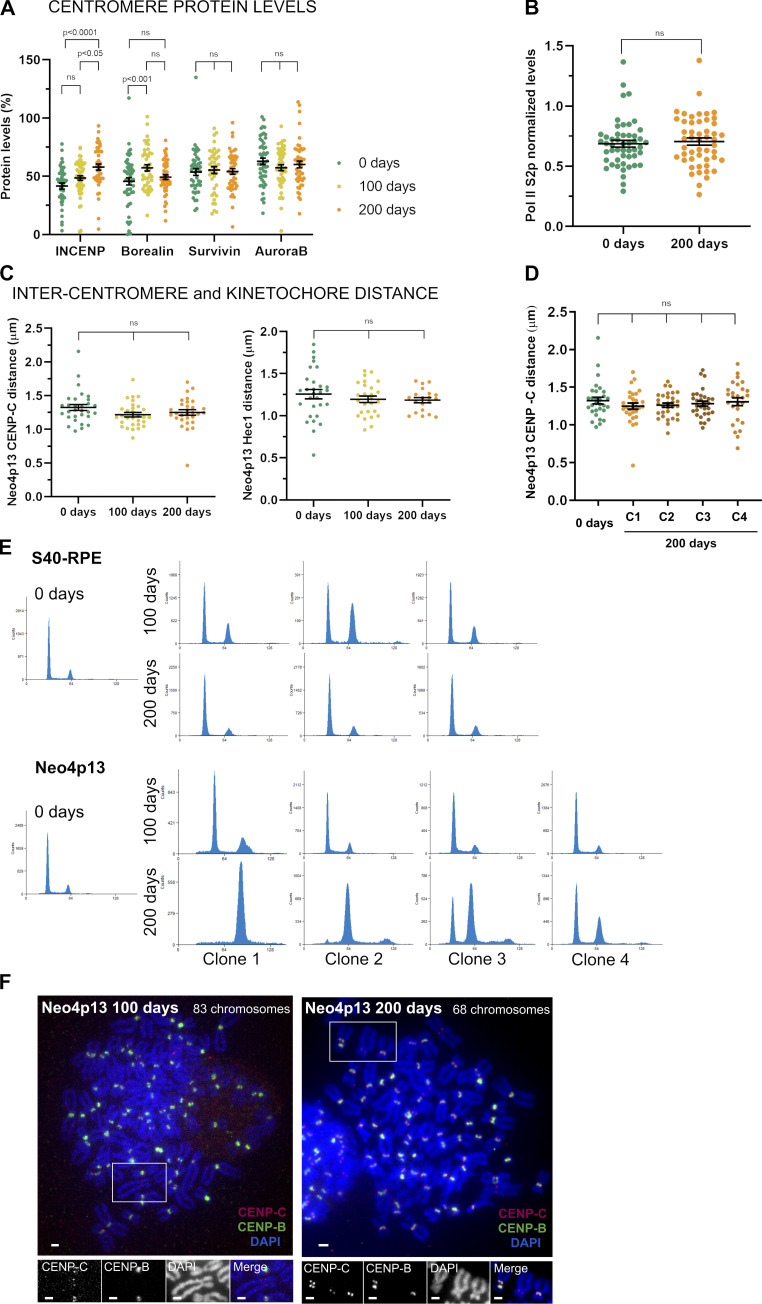
**The neocentromere adapts by accumulating INCENP and Borealin. (A)** CPC component protein levels at Neo4p13 at indicated times points (0, 100, and 200 d of continuous culture) measured as in Fig. 2 A. Mean and SEM of five independent experiments (*n* = 47–54 spreads). P values are based on one-way ANOVA with Tukey’s multiple comparison test. **(B)** Pol II S2p levels at Neo4p13 at 0 and 200 d of continuous culture measured as in Fig. 3 E. Mean and SEM of five independent experiments (*n* = 50 spreads). P values were determined as in A. **(C)** Intercentromere and inter-kinetochore distance at Neo4p13 at indicated time points based on CENP-C and Hec1 immunostaining measured as in Fig. 2 C. Mean and SEM of three independent experiments (*n* = 20–32 spreads; P values are indicated in the figure, determined as in A). **(D)** Intercentromere distance based on CENP-C of different clones of Neo4p13 (C1–C4, four independent clones) after 200 d in continuous culture measured as in Fig. 2 C. Mean and SEM of three independent experiments (*n* = 27–30 spreads). P values are defined as in A. **(E)** Cell cycle profiles determined by flow cytometry using PI DNA staining of S40-RPE and Neo4p13 at 0, 100, and 200 d of continuous culture. Three independent populations for S40-RPE and four for Neo4p13 subjected to long-term culture experiments are shown. **(F)** Representative spreads of polyploid cells of Neo4p13 cell line after 100 or 200 d in continuous culture, stained for indicated proteins and DNA (as for Fig. 2 B). A neocentromere-containing chromosome is identified by lack of CENP-B staining. Scale bars, 2 µm.

As Neo4p13 is generated experimentally, it allows us to time stamp and track the fate of this young neocentromere through maturation. We monitored neocentromere composition and chromatin status over the course of 200 d of continuous culture, equating roughly 200 divisions (∼1.5 × 10^60^ cells; Fig. 5 A). If we assume that there are 4 × 10^13^ cells in the human body (Bianconi et al., 2013), then during our experimental time frame, we subjected the Neo4p13 line to >4.5 times more cell divisions than required during human embryonic development. Most centromere components maintain levels comparable to those observed shortly after centromere formation. Interestingly, we detected a significant gradual increase of INCENP levels (Fig. 5 B), as well as Borealin, albeit to a lesser extent (Fig. S3 A). The gradual increase of INCENP is of interest, as INCENP directly contacts chromatin (Jeyaprakash et al., 2007; Klein et al., 2006; Serena et al., 2020). It is possible that chromatin structure is changing through successive divisions driving more INCENP to accumulate. To extend this observation, we analyzed the centromere status of several independent clones of the Neo4p13 after 200-d culture. Inner-centromere maturation is a robust phenotype, as we measured a significant increase in INCENP levels in three out of four long-term cultures, accompanied by a significant increase in Borealin levels in one of the clones (Fig. 5 C). Conversely, CENP-C levels decrease over time, suggesting that either changes in chromatin structure or increased CPC recruitment may compete with CENP-C binding.

Further, we find that transcription is significantly increased after 200-d culture (Fig. 5 D), indicating a more active chromatin state, correlating with decreased heterochromatin (see below), although levels of Pol II S2p remained unchanged (Fig. S3 B). The low initial cohesin levels are also maintained during the 6 mo of culturing (Fig. 5 E), correlating with the lack of any major changes in inter-kinetochore distance (Fig. S3, C and D). Further, H3K9me3 levels gradually decrease over time but do so across a broad domain extending almost 4 Mb around the neocentromere. Although possibly linked to the neocentromere, it is perhaps more likely that this is a consequence of a systemic effect of long-term culture (Fig. 5 F). The diminishment of H3K9me3 is of relevance, as it was shown that pericentric heterochromatin loss affects the recruitment of centromere and kinetochore components (including CPC) and increases intercentromere distance (Molina et al., 2016a). Thus, the H3K9me3 loss may explain the lack of improvement in intercentromere distance over time. Taken together, our results indicate that once formed, the neocentromere is stably transmitted through many mitotic divisions and matures by restructuring chromatin, particularly at the inner centromere.

It has been proposed that over the course of evolution, neocentromeres can acquire α-satellite DNA (evolutionary new centromeres; Rocchi et al., 2012; Tolomeo et al., 2017). Therefore, we extended our nanopore analysis to cells following 200 d of continuous culture. Neo4p13 cells after long-term culture showed no new insertions in the neocentromere region (Fig. S2 B). Interestingly, after 200 d, we no longer detected reads from the circular DNA bearing the deleted satellite-containing endogenous centromere (Fig. S2 C), indicating that is lost following prolonged culture.

Finally, we found that due to long-term culture, the cell population showed a gradual increase in ploidy. Changes in ploidy may be a mere consequence of SV40 expression. However, long-term culture of Neo4p13 showed increased levels of polyploidization compared with S40-RPE, despite that both cell lines expressed SV40 (Fig. S3 E). The degree of polyploidization across parallel cultures of Neo4p13 was also variable (Fig. S3 E). This may be due to selection and extended culturing, but we cannot discard that the neocentromere induces a small rate of missegregation leading to different levels of ploidy over long-term culture. Importantly, even in the polyploid state, cells maintain the neocentromere (Fig. S3 F), indicating mitotic stability even in the absence of selection pressure to maintain chromosome 4.

In sum, we report a methodology for centromere deletion and neocentromere isolation. We characterize a novel human neocentromere derived from cultured cells and discovered that the nonsatellite-based neocentromere shows specific defects in inner-centromere structure that adapts during successive culturing. Our method paves the way for isolation of additional neocentromeres to derive general principles of centromere specification.

## Materials and methods

### Cell line and culture conditions

The human cell line used was derived from hTERT RPE-1 cells (ATCC; CRL-4000). Detailed steps for system construction and targeted cassettes are outlined in Fig. S1 A. eYFP/Puromycin expression cassettes as outlined in Fig. S1 were targeted by adeno-associated virus (AAV)–mediated delivery (Berdougo et al., 2009; Mata et al., 2012). For this, targeting constructs were cloned into NotI site of pAAV-LacZ and transfected along with pAAV-RC and pHELPER (all plasmids from Agilent; AAV Helper-Free System, catalog no. 240071) into HEK293 cells (ATCC; CRL-1573). Virus was harvested, RPE target cells were infected, and targeted clones were selected by FACS 4 d after infection. Clones were further expanded and resorted for monoclonal lines. Cre recombinase was expressed by transfection of pCAGGS-nlCre, a vector expressing an optimized Cre recombinase containing an N-terminal nuclear localization signal (Khandelia et al., 2011). We aimed to reduce the DNA damage response and maintain cell viability following Cas9 expression (parental cell line, S40-RPE). To this end, we expressed the oncogenic virus SV40 large T-antigen (Hermannstädter et al., 2009; Lin and Simmons, 1991) by electroporation (pcDNA3.1 plasmid expressing SV40 large T-antigen and conferring resistance to neomycin, a gift from Colin Adrain, Instituto Gulbenkian de Ciência [IGC], Oeiras, Portugal) and selected expressing clones with 100 µg/ml of G418 Geneticin. Cells were grown at 37°C, 5% CO_2_ in DMEM/F-12 cell culture media. Media was supplemented with 10% FBS, 2 mM glutamine, 14.5 mM sodium bicarbonate, 100 U/ml penicillin, 100 µg/ml streptomycin, and 1% nonessential amino acids.

Electroporations for Cas9/gRNA delivery were performed using the Neon Transfection System (Invitrogen) according to the manufacturer’s instructions. We used the pX330-U6-Chimeric_BB-CBh-hSpCas9 plasmid (Addgene; catalog no. 42230) in which we cloned either gRNA1 (5′-GCA​CCC​CTG​CTG​GGA​GGG​TT-3′) or gRNA2 (5′-GAA​CCA​AAC​CCT​CCC​AGC​AG-3′), both targeting the mouse intronic region (see Fig. S1). Typically 4 × 10^6^ cells were electroporated with a total of 10 µg plasmid DNA cocktail, two time pulses of 20 ms at 1,100 V and 1,350 V combined. After 72 h, cells were subjected to cell sorting. Note that while centromere deletion and chromosome arm ligation reconstitutes a Puromycin resistance cassette, Puromycin selection was not used in our experiments.

### Microscopy

Images were collected at room temperature on a Deltavision Core system (applied precision) inverted microscope (Olympus; IX-71) coupled to a Cascade II 2014 Electron Multiplying Charge-Coupled Device (EM-CCD) camera, using a 100× 1.4 NA oil-immersion objective, or a Leica High Content Screening microscope, based on the Leica DMI6000 equipped with a Hamamatsu Flash Orca 4.0 sCMOS camera using a 100× 1.44 NA objective, controlled with Leica LAS X software. Images were collected as 0.2-µm z sections. Images presented in figures are maximum intensity projection. For Fig. 1 E, images were deconvolved with Applied Precision's softWorx software. Fluorophores imaged are those conjugated to secondary antibodies, listed below.

### Mitotic spreads

Mitotic spreads were prepared after mitotic shake-off of cells arrested ∼6–7 h in 100 ng/ml Gibco KaryoMAX Colcemid Solution in HBSS. Cells were incubated in 5% vol/vol FBS and 0.5% wt/vol sodium citrate for 5–10 min and subsequently transferred to a coverslip containing a film of fixative (1% vol/vol formaldehyde and 0.5% vol/vol Triton X-100 in milliQ water, pH 9.2 titrated with borate solution). Coverslips were left horizontally overnight in a humid chamber at RT and air dried for a few minutes before processing for IF or FISH-IF.

### IF

The IF procedure was based on Bodor et al. (2012). Coverslips fixed with 1% formaldehyde (see Mitotic spreads) were transferred to a parafilm-covered glass plate in a humid dark box and blocked for 30 min at 37°C in IF blocking buffer (2% [vol/vol] FBS, 2% [wt/vol] BSA, 0.1% [vol/vol] Triton X-100, and 0.04% [wt/vol] NaN3 in 1× PBS). Cells were incubated with primary antibody diluted in IF blocking buffer for 60 min at 37°C. Coverslips were washed three times for 5 min at room temperature in 1× PBS containing 0.1% (vol/vol) Triton X-100 and incubated with secondary fluorescent antibody diluted in blocking buffer for 30 min at 37°C. Coverslips were washed three times again, incubated with DAPI (Sigma), washed with 1× PBS, and mounted in Mowiol for imaging or post-fixed for FISH (see below).

#### Primary antibodies used for staining

Aurora B (at concentration 1:200) from BD Transduction Laboratories; INCENP (1:200) from Abcam (ab23956); Survivin (1:250) from Novus Biologicals (NB500-201 lot:AB-1); Borealin (1:500) from MBL 1D11 (m147-3 lot:012); CENP-B 1:600 from Abcam (ab25734) or 1:400 for (ab167361); CENP-C 1:150 from Hybridoma clone LX191 (from Don Cleveland, University of California, San Diego, San Diego, CA) or 1:500 for MBL International PD030; CENP-H (1:150; from Song-Tao Liu, University of Toledo, Toledo, OH); CENP-T (1:500) from Covance (Don Cleveland, University of California, San Diego, San Diego, CA); ACA (1:150) from Antibodies Incorporated 15–234; Sgo1 (1:200) from Abcam (ab58023); H3T3ph (1:50) from Cell Signaling (#9714); H2AT120ph (1:100) from Active Motif (39391); Bub1 (1:100) from Abcam (ab54893); Zw10 (1:200) from Abcam (ab21582); Hec1 (1:200) from Thermo Fisher (MA1-23308); and Pol II S2p (1:250) from Abcam (ab252855).

#### Secondary antibodies

FITC-conjugated anti-rabbit (611–702-127), Texas Red–conjugated anti-mouse (610–109-121), anti-rabbit (611–108-122), and FITC-conjugated anti-guinea pig (606–102-129) were all obtained from Rockland Laboratories. FITC-conjugated anti-mouse (715–095-151), FITC-conjugated anti-rat (712–095-153), and TRITC-conjugated anti-rabbit (111–025-144, for Rhodamine channel in Leica microscope) were obtained from Jackson ImmunoResearch. Dye680LT-conjugated anti-human antibody was from LI-COR (926–68032). All were used at 1:400.

### FISH

For the FISH procedure, coverslips processed for IF were post-fixed with 1% PFA in 1X PBS for 10 min, quenched 5 min in 0.1 M Tris, pH 7.4, and incubated in 1X PBS for 5 min. Coverslips were washed twice with 2X SSC followed by incubation with RNase (100 µg/ml in 2X SSC) for 30 min, HCl (0.1 M) for 10 min, and 0.5% saponin, 0.5% Triton in PBS 1X for 10 min before denaturation in 70% formamide 2X SSC for 3 min at 75°C.

The FISH probe, denatured for 5 min at 75°C, was added onto the coverslip, placed onto a slide, sealed with rubber cement, and incubated overnight at 37°C. Next, coverslips were washed with 2X SSC for 5 min at RT, 1X SSC for 2 min at 75°C, 2X SSC + 0.05% Tween for 3 min at RT, and 2X SCC and then DAPI stained and mounted.

FISH probe against the chromosome 4 arm was generated by Nick Translation synthesis, labeled with tetramethylrhodamine-5-2′-deoxy-uridine-5′-triphosphate (Roche), and resuspended in 50% formamide in 2X SSC, 10% dextran sulfate, and 0.1% Triton X-100. An equimolar mixture of the following BACS (BAC PAC Resources Center, Oakland, CA) were used as template for the reaction (RP11-33C2, RP11-1113G13, RP11-204I22, RP11-209G6, RP11-120B2, RP11-626J9, RP11-1144D2, RP11-235H8, and RP11-70M20 [covering the 4q21 locus]).

### mFISH karyotyping

Cells grown to ∼75–80% confluency were treated with 100 ng/ml Colcemid (Roche) for 3 h and prepared as described previously (Trott et al., 2017). Briefly, mitotic cells were collected by mitotic shake-off after a short trypsin treatment and centrifuged at 1,000 rpm for 10 min. Cell pellets were resuspended in 75 mM KCl and incubated for 15 min in a 37°C water bath. Carnoy fixative solution (methanol/acetic acid 3:1) was added to the cells at 1/10 volume before centrifugation at 1,000 rpm for 15 min. Cells were then fixed 30 min at room temperature in the Carnoy solution, centrifuged and washed once more with fixative. A minimal volume of fixative was left to resuspend the pellet and cells were dropped onto clean glass slides. mFISH staining was performed following manufacturer’s instructions (MetaSystems). The Metafer imaging platform (MetaSystems) and Isis software were used for automated acquisition of the chromosome spread and mFISH image analysis. The mixture of colors within individual chromosomes in some of the karyotypes (see, for example, Fig. 1 chromosome 2 in Neo4p13) is due to physical overlap within the spreads and does not represent translocation or fusion events.

### Quantification of centromere intensities

For centromeric proteins quantification of microscopy images, centromere signals of adjacent sisters of neocentromere and random centromeres were measured in Fiji (ImageJ) by manual quantification using a region of interest box of 10 × 18 pixels accounting for background correction. Specific chromosomal markers were used as described in the text to detect centromeres of interest and signals were normalized within each cell spread. For centromere distance, single-plane images containing in focus signal for neocentromere and random canonical centromeres were selected, and in Fiji (ImageJ) a straight line intersecting with both centromeric dots (for CENP-C or Hec1) was plotted, and the distance between the maximum intensity peak of each dot pair was measured. For each measurement, three to five independent experiments were performed per condition, quantifying 28–52 independent spreads (*n*). The mean and SEM were determined and plotted. Data statistics were analyzed in GraphPad Prism using one-way ANOVA and the multiple comparison Tukey test to compare simultaneously all pairwise comparisons and identify any difference between two means that is greater than the expected standard error. We selected this method in order to account for the variability among canonical centromeres in the significance test. The P values are indicated within the figures. The only exceptions were for Fig. 4 A, where we performed a *t* test analysis for the Sgo1 data, and Figs. 3 D and 5 D, where we performed multiple *t* test or a two-way ANOVA, respectively, for the quantitative PCR data (indicated in the figure legend).

### Cell sorting and flow cytometry

Cell suspensions were sorted on a MoFLo high speed cell sorter (Beckman Coulter), equipped with a 488-nm laser used for scatter and autofluorescence measurements and a 514-nm laser for eYFP excitation. The instrument was run with a 100-µm nozzle, and a Forward Scatter Neutral Density Filter UV2.0 was added to improve eYFP detection. Cells were collected into conditioned medium (50% 0.45-µm filtered medium collected from cultured cells and 50% fresh medium supplemented with 20% FBS and antibiotics/antimycotics [Fungizone at 250 µg/ml and Gentamicin/Amphotericin B (Gibco) at 10 µg/ml and 250 ng/ml final concentration, respectively]) maintained at 4°C.

For cell cycle analysis, cells were harvested and fixed for 30 min at 4°C with 70% ethanol. Cells were washed in PBS and incubated for 1–3 h at 37°C with RNase A in PBS (100 µm/ml). Finally, cells were resuspend in 50 µm/ml propidium iodide (PI; Sigma) in PBS. Subsequent flow cytometry analysis was performed on a BD Fortessa X20 (Becton Dickinson) using Diva software.

### ChIP

#### CENP-A native ChIP

10 × 10^6^ cells were collected and equilibrated in ice-cold CIB buffer (3.75 mM Tris, pH 7.5, 20 mM KCl, 0.5 mM EDTA, 0.5 mM DTT, 0.05 mM Spermidine, 0.125 mM Spermine, 0.1% IGEPAL, 1 mM PMSF, 0.1% aprotinin [0.3–2 Trypsin Inhibitor Unit], and one tablet Complete protease inhibitor cocktail [Roche; 11836153001] per 10 ml) and homogenized 10 times with a Douncer with a tight pestle. Cells were centrifuged at 300 *g* for 5 min at 4°C, and the Dounce homogenization was repeated. Pelleted nuclei were washed with 15 ml washing buffer A (20 mM Hepes sodium, pH 7.5, 20 mM KCl, 0.5 mM EDTA, 0.5 mM DTT, 0.5 mM PMSF, 0.1% aprotinin, and Complete protease inhibitor cocktail) and centrifuged at 300 *g* for 5 min at 4°C. Washing was repeated with 7.5 ml washing buffer A supplemented with 0.3 M NaCl and centrifuged at 500 *g* for 10 min at 4°C. Pelleted chromatin was resuspended in washing buffer A and digested with micrococcal nuclease (NEB; 2,000 U) in the presence of 0.3 M NaCl and 3 mM CaCl_2_ while rotating for 1 h at RT. The reaction was quenched with 5 mM EGTA and 0.05% NP-40 and centrifuged at 10,000 *g* for 15 min at 4°C. Input sample was taken, and the remainder was divided in two aliquots, a control immunoprecipitation (no antibody added) and an immunoprecipitation to which CENP-A antibody (8 µg from clone A5 from Ando et al. [2002]) or Abcam (ab13939) was added and incubated at 4°C, rotating overnight. Immunocomplexes were recovered by addition of 75 µl protein G magnetic beads (Dynabeads) washed with buffer B (20 mM Hepes sodium, pH 7.5, 20 mM KCl, 0.5 mM EDTA, 0.5 mM DTT, 0.5 mM PMSF, 0.1% aprotinin, 0.5% NP40, 0.5 M NaCl, and Complete protease inhibitor cocktail) and incubated 4 h at 4°C while rotating. Samples were washed two times with washing buffer B and then recovered in 250 µl TNES buffer (10 mM Tris, pH 7.5, 300 mM NaCl, 10 mM EDTA, and 1% SDS) with 100 µg/ml RNase A. For the input samples, an equal volume of TNES with RNase A was added. Samples were incubated for 15 min at room temperature, followed by proteinase K (20 µg/ml) addition and further incubation for 1 h at 50°C. Finally, samples were subjected to a phenol-chloroform extraction followed by purification on a Qiagen column (MinElute PCR cleaning columns).

#### Cross-linked ChIP

For cross-linked ChIP, 8 × 10^6^ cells were collected, either random cycling or enriched in mitosis by incubation of cells for 24 h with 2.4 µM Eg5 inhibitor III (Calbiochem; Dimethylenastron-DMEIII), depending on the experiment (indicated in each figure). Cells were then cross-linked with 1% formaldehyde for 8 min and quenched with 125 mM Glycine. Cross-linked cells were resuspended in SDS lysis buffer (50 mM Tris, pH 7.5, 1% SDS, and 10 mM EDTA) supplemented with 2 mM PMSF and Complete protease inhibitor cocktail for sonication. For quantitative ChIP, 1% cross-linked immortalized mouse embryonic fibroblasts (gift from Colin Adrain, IGC) were added to each sample. Sonication was performed using a QSonica Q800R3 Sonicator for 3.5 min (30 s on/off cycles) at 50% amplitude, shearing DNA to an average size of ∼0.8 kb. For ChIP, sonicated chromatin was diluted five fold in ChIP dilution buffer (20 mM Tris, pH 7.5, 0.1% SDS, 1% Triton X-100, 150 mM NaCl, and 2 mM EDTA) supplemented with Complete protease inhibitor cocktail. Chromatin was centrifuged 10 min at 10,000 *g* at 4°C, and the supernatant was incubated overnight with 6–8 µg of the appropriate antibody along with 25 µl of protein G magnetic beads (Dynabeads) washed with ChIP dilution buffer (the input sample was isolated before). Antibodies used were anti-H3K9me3 (Abcam; ab8898), anti-Rad21 (Abcam; ab992). ChIP washes were performed at 4°C (twice with each buffer), and all buffers were supplemented with Complete protease inhibitor cocktail consisting of a low-salt wash buffer (50 mM Tris, pH 7.5, 150 mM NaCl, 1 mM EDTA, 1% Triton X-100, and 0.1% Na-Deoxycholate), high-salt wash buffer (50 mM Tris, pH 7.5, 500 mM NaCl, 1 mM EDTA, 1% Triton X-100, and 0.1% Na-Deoxycholate), LiCl wash buffer (10 mM Tris, pH 7.5, 0.5% NP-40, 0.5% Na-Deoxycholate, 250 mM LiCl, and 1 mM EDTA), TE buffer (10 mM Tris, pH 7.5, and 1 mM EDTA), and Tris pH 7.5 buffer. Beads (or input) were resuspended in 30 µl tagmentase reaction buffer (10 mM Tris Cl, pH 8.0, and 5 mM MgCl_2_) and 1 µl TDE1 (Tn5 enzyme) from the Nextera DNA library kit (Illumina) and incubated for 10 min at 37°C on a Thermomixer (1,200 rpm). Washes with LiCl buffer and TE buffer were performed on the beads before SDS elution buffer (same as lysis buffer) was added to both the input sample and beads. Samples were incubated at 65°C overnight to decrosslink DNA from proteins and then treated with RNase and proteinase K and processed for DNA purification as previously indicated for native ChIP.

### Next-generation sequencing

#### CENP-A native ChIP

Sequencing libraries were generated and barcoded for multiplexing. For library preparation, we used NEBNext Ultra II DNA library prep kit for Illumina (E7645) and NEBnext Multiplex oligos for Illumina (NEB; E7335) following the manufacturer's recommendations. Libraries were size selected using AMPure XP magnetic beads (Beckman Coulter) to exclude polynucleosomes (≥220 bp) and PCR amplified before submission to 75-bp single-end sequencing on Illumina NextSeq 500 platform.

#### Cross-linked ChIP

Sequencing libraries were generated and barcoded for multiplexing using adaptors described previously (Buenrostro et al., 2013). The average size and concentration of all libraries were analyzed using the Fragment Analyzer system (Agilent) followed by quantitative PCR using KAPA Library Quantification Kit (Illumina). Libraries were sequenced as 75-bp single-end reads on an Illumina NextSeq 500 platform.

### ChIP-seq data processing

All data were processed on the Galaxy platform (usegalaxy.org).

#### CENP-A native ChIP

Single-end ChIP-seq reads were aligned to the human genome build hg38 with BWA (Galaxy tool version 0.7.17.4). Duplicate-read removal was performed by using the RmDup (Galaxy tool version 2.0.1 tool version 1.3.1). BamCoverage and BamCompare deeptools were performed with 1,000 bin size and read number normalization. Plots were generated using Integrative Genomics Viewer (Robinson et al., 2011).

#### Cross-linked ChIP

For quantitative (calibrated) ChIP analysis, reads were analyzed following Hu et al. (2015). Reads were trimmed (version tool 1.0.2) to keep bases 11 to 75, and any reads smaller than 50 bp were removed (FASTQ filter tool) to minimize genome misalignments. Reads were aligned to the human genome build hg38 and the mouse genome build mm10 using Bowtie 2 with the very sensitive end-to-end preset option. Output fasta files of aligned and unaligned reads were generated. Unmapped reads against mm10 were aligned against hg38. To internally calibrate the ChIP-seq, the exogenous mouse genome spike-in was used to quantitatively compare the genomic profiles of chromatin modifications or protein binding profiles between experimental conditions as described previously (Hu et al., 2015; Orlando et al., 2014; Bonhoure et al., 2014). To account for any variation in spike-in cell mixing in different samples, the factors were corrected using the read counts corresponding to input samples as described previously (Fursova et al., 2019). BamCoverages (deeptool) normalized for read number and including the appropriate scaling factor were loaded into Integrative Genomics Viewer for visualization.

As in Neo4p13, signals are coming from both chromosome 4 homologues (one carrying the neocentromere), we estimated the coverage for H3K9me3 or Rad21 over the 4p13 region coming from the neocentromere allele. For this estimate, we assumed the S40-RPE signal is originating equally from both alleles and that the nonneocentromere chromosome 4 in Neo4p13 is equivalent to both S40-RPE chromosomes 4. Quantitative data of the number of reads per 10,000 kb of chromosome 4 was normalized against S40-RPE, and a further normalization step (1 = maximum level and 0 = minimum value) for equal scaling was performed.

The data reported in this paper were deposited in the Gene Expression Omnibus database (accession no. GSE155829 for the SuperSeries composed of the following SubSeries: GSE155826 for native CENP-A ChIP-seq and GSE155828 for quantitative ChIP-seq results [H3K9me3 and Rad21]).

### Nanopore sequencing and data processing

Genomic DNA was extracted using a Qiagen Blood & Cell culture DNA Midi kit following the manufacturer's recommendations. Libraries were prepared using the LSK109 one-pot kit, and one flow cell was run on the PromethION for each sample. Reads were base called with the guppy 3.0.5 high-accuracy model and initially mapped with minimap2 v 2.14 to the hs37d5 human reference genome.

For the detection of structural variants, Sniffles was run genome-wide on each sample bam file with the parameters -l 10 -s 5 (minimum length of 10 and minimum read support of 5). Calls were initially filtered using tabix to select those within or overlapping the region spanning chromosome 4 (40,000,000–60,000,000). Parental calls were subtracted from the ones corresponding to Neo4p13 neocentromere 0 d and 200 d to select for structural variants that were only present in the latter two samples. For this subtraction, two calls were considered the same if they were of the same structural variantion type and if their start positions and SV lengths were both within a certain distance of each other. This distance, x, was specified as x=5 if parental SV length <250, or x = parental SV length / 50 otherwise. Any remaining calls within the region spanning the neocentromere area (chromosome 4: 43,000,000–43,250,000) were individually examined.

Analysis of structural variants within the S40-RPE and Neo4p13 region revealed no evidence of translocations to the hs37d5 decoy chromosome (an artificial in silico chromosome containing all human genome sequences that are not mapped in the reference genome), indicating no insertion of α-satellite sequences or any other. Overall, our analysis of the 4p13 region in the Neo4p13 line failed to show any change in the DNA composition compared with S40-RPE with a 10-bp detection cutoff.

For de novo assembly of the parental and neocentromere consensus haplotypes, reads mapping to 4:40,000,000–60,000,000 in the parental sample were extracted from the bam file and used to build a de novo assembly of the region. This assembly was made using Flye v2.5 (https://github.com/fenderglass/Flye), with parameters -g 20–assm-coverage 40. The assembly was followed by three rounds of polishing with Racon v1.4.3 (https://github.com/isovic/racon) and consensus correction using Medaka v 0.8.1 (https://github.com/nanoporetech/medaka) with the r941 prom high model. Racon parameters were chosen as recommended for use with Medaka (-m 8 -x -6 -g -8 -t 4 -q -1 -w 500). This assembly included one contig spanning 4: 39,982–49,117 kb and a second spanning 4: 51,794–58,879 kb. The former included the cassette insertion, while the latter represented the unmodified haplotype.

Reads from the parental and neocentromere 0-d and 200-d samples were remapped to the corrected parental assembly, and sniffles was run as before. Variant Call Format files were examined for calls within the region of the contig mapping to 4:43,000,000–43,250,000. De novo assemblies of the deleted haplotype in the neocentromere samples were made by removing reads covering the sites 50 bp inside each end of the deletion from the bam files, extracting the fastqs, and running these through the same Flye–Racon–Medaka pipeline as described above. In each case, the assemblies included a single contig covering the cut sites and at least a couple of megabases on either side.

### RNA extraction and quantitative RT-PCR

RNA was isolated using Trizol reagent (Life Technologies). Cells were lysed by pipetting and incubated for 5 min at RT. Next, 0.2 ml chloroform was added per sample, mixed, and incubated for 3 min at room temperature followed by centrifugation at 12,000 *g* for 15 min at 4°C. The aqueous phase was mixed with 0.5 ml of 100% isopropanol and incubated at RT for 10 min followed by centrifugation at 12,000 *g* for 10 min at 4°C. The supernatant was removed and the pellet was washed with 1 ml of 75% ethanol and air dried for 10 min. Next, the RNA pellet was resuspended in 100 µl of RNase-free water and incubated at 56°C for 10 min. The residual DNA was removed with DNase I (RNase-free) from NEB at 30 C for 30 min before purification with RNeasy Mini kit (QIAGEN) following the manufacturer’s instructions. cDNA was prepared from 2 µg RNA using a High-Capacity RNA-to-cDNA Kit (Applied Biosystems), and the libraries were diluted five times before quantitative PCR measurements. RNA was isolated from independent biological replicates, and RT-PCR runs were performed in triplicate for each sample using SYBR Green Supermix, iTaq Universal (Bio-Rad) in a CFX384 Touch Real-Time PCR Detection System (Bio-Rad). The quantitative PCR conditions were 95°C for 3 min (95°C for 10 s; 59°C for 30 s) × 52 cycles using the following primers (300 nM final concentration, sequence indicated 5′–3′): SNRPD3_F (GAG​GGC​CAC​ATT​GTG​ACA​TGT​G), SNRPD3_R (GGC​AGT​TCA​TGT​TGT​CCT​CTG​C); Post_4p13_F (GCT​GCA​GCG​GCC​TGT​AAC​CT), Post4p13_R (CTC​CAT​TGT​CCC​CGT​GCG​CA); 4p13-1_F (CCT​GAT​GCC​AAT​TCC​CAC​GGA​GG), 4p13-1_R (ACC​TCC​AGG​GAC​ACA​GTT​CAA​GC); 4p13-2_F (TGC​ACC​CAA​CAC​TGG​AGC​ACC), 4p13-2_R (TCT​GCG​CAA​ATG​GGG​TTT​TGA); 4p13-3_F (TGG​CCC​CAG​GCT​CTG​TCA​GT), 4p13-3_R (TCA​AGC​CCG​AGA​CCT​GGC​AA); and GRXCR1_F (AGA​AGG​GCC​GAG​TGG​GAG​CA), and GRXCR1_R (TGT​GCC​CAG​AGC​CGT​CTC​CA).

### Online supplemental material

Fig. S1 illustrates the gene-targeting strategy and genomic architecture of the cell line created to induce centromere deletion, as well as detection of the neocentromere in early isolates and karyotyping of neocentromere-containing cells and alternative survivors. Fig. S2 shows AT content across chromosome 4, long-read sequencing plots to detect DNA sequence changes within the neocentromere locus, and genomic views of H3K9me3 and Rad21 occupancy. Fig. S3 shows inner-centromere protein and RNA polymerase occupancy as well as measures of intercentromere distance in long-term culture.
